# The Effect of Educational Intervention Based on Health Belief Model on Eye Care Practice of Type II Diabetic Patients in Southern Iran

**DOI:** 10.1155/2022/8263495

**Published:** 2022-08-22

**Authors:** Babak Pezeshki, Golnoosh Karimi, Fatemeh Mohammadkhah, Pooyan Afzali Harsini, Ali Khani Jeihooni

**Affiliations:** ^1^Department of Internal Medicine, School of Medicine, Fasa University of Medical Sciences, Fasa, Iran; ^2^Department of Public Health, School of Health, Fasa University of Medical Sciences, Fasa, Iran; ^3^Department of Community Health, Child Nursing and Aging, Ramsar School of Nursing, Babol University of Medical Sciences, Babol, Iran; ^4^Department of Public Health, School of Health, Kermanshah University of Medical Sciences, Kermanshah, Iran; ^5^Nutrition Research Center, Department of Public Health, School of Health, Shiraz University of Medical Sciences, Shiraz, Iran

## Abstract

**Background:**

The ocular complication caused by diabetes is one of the most common reasons of blindness in the world. This study aimed to investigate the effect of educational intervention on eye care practice of type II diabetic patients based on health belief model (HBM) in Fasa city.

**Methods:**

This study was a quasi-experimental study on 100 patients with type II diabetes referred to the diabetes center in Fasa city, Fars province, Iran, in 2019. Data were collected using a valid self-reported questionnaire including demographic variables, knowledge, and HBM (perceived susceptibility, perceived severity, perceived benefits, perceived barriers, self-efficacy, and cues to action), and eye care performance (based on self-report) and the level of HbA1cof both groups were measured before and three months after the educational intervention. The experimental group received training in eight sessions; each session lasted for 50 to 55 minutes. In order to analyze the studied data, SPSS 22 software (SPSS Inc., IBM, Chicago, IL, USA), Chi-square, independent *t*-test, and paired *t*-test have been used. *P* < 0.05 was considered as statistically signiﬁcant.

**Results:**

The results showed that the mean scores of knowledge (*P* < 0.001) and HBM components (*P* < 0.001) in the experimental and control groups after intervention have a signiﬁcant difference. After the training program, eye care performance in the experimental group was better than that in the control group (*P* < 0.001). Furthermore, HbA1c (*P* < 0.001) improved signiﬁcantly in the experimental group compared to the control group.

**Conclusions:**

Planning and implementing education using the HBM to improve eye care performance in diabetic patients are very effective and beneﬁcial. Moreover, educational programs based on health education and health promotion models for diabetic patients for preventing side effects caused by diabetes should be performed.

## 1. Background

Diabetes is a metabolic disease and multifactorial disorder which is specified by chronic enhancement of blood glucose level or hyperglycemia, and it is caused by the disorder of secretion or insulin function or both of them. Diabetes is called the silent epidemic disease, and it is a general health problem which is responsible for 9% of all deaths around the world. Diabetes accompanies various disorders in glucose level, protein, and oil metabolisms. The chronic enhancement of blood glucose level causes functional problems and failure in different body organs, especially eyes, kidneys, nerves, and cardiovascular problems [1]. Nowadays, diabetes is the fifth reason of death in western societies and the fourth common reason of people's reference to health centers [2]. As a silent disease, diabetes is one of the serious global problems. It has been revealed that, in 2010, there were 285 million adult diabetic patients around the world [3]. It was predicted that, by 2021, more than 12 million people would be diagnosed by diabetes. In Iran, the costs related to diabetes are almost 590.676 million dollars annually [4]. The costs and disablement caused by this disease are very high, and it is one of the most common health issues [5]. The prevalence of this disease in Iran is almost 6% of population [6]. One of the side effects of diabetes is optical problems [7]. The number of persons with visual impairment due to diabetic retinopathy worldwide is rising and represents an increasing proportion of all blindness/severe vision impairment causes. In sub-Saharan Africa and South Asia, age-standardized prevalence of diabetic retinopathy-related blindness and severe vision impairment was higher. 1 out of 52 visually impaired people had visual impairment due to diabetic retinopathy, and one out of 39 blind people had blindness due to diabetic retinopathy [8]. Diabetic patients must control their blood glucose level to prevent the optical problems, and this issue is only possible by awareness and proper self-care practice of patients [9].

The general idea is that, in order to recognize their disease and healthcare measures and change their health behaviors, patients need education and help [10].

The efficiency of education depends on the proper usage of theories of behavioral sciences [11].

One of the efficient models of health education is health belief model which states that the behavior of a person depends on the knowledge and perspective of that person. This model also causes people to understand health threatening issues and helps them to adopt appropriate behaviors to have a healthy life [12].

The reason of using this model is investigating the reasons of rejecting health issues by people and explaining behavior of people who think that they will never get infected by disease [13]. The results of other studies indicated that, using health belief model about self-care behaviors was very efficient among type II diabetic patients [14, 15]. In diabetic patients, it is not possible to have constant access to the doctor and health staff. A major part of the treatment is done by the patient himself. Therefore, self-care training is very necessary to control and reduce the complications of the disease [16]. On the other hand, according to those studies, the probability of doing the recommended behavior by the person increases when she/he perceives her/himself as susceptible to the disease; the disease is serious, know the benefits of health behavior, have few barriers to behavior, and have cues to actionfor performing health behavior [17].

Due to the increase in the incidence and prevalence of diabetes, according to the mentioned issues, considering the characteristics and effectiveness of interventions based on the health belief model in behavior change, and due to the importance of eye care in diabetic patients, the purpose of the current study is investigating the effect of educational intervention on eye care practice of type II diabetic patients based on health belief model.

## 2. Methods

The present study is a quasi-experimental study conducted in 2019. The studied subjects are patients suffering from type II diabetes who were referred to the diabetes center (governmental and referral) of Fasa city, Fars province, in the south of Iran. Initially, based on the study criteria, 200 patients with type II diabetes having medical file in the diabetes center of Fasa city have been invited to participate in the study programs. The inclusion criteria were to be 30 years of age and older and have a history of diabetes disease. Some of the patients discontinued the study. The exclusion criteria were unwillingness to continue participating in the study, getting infected by optical and cardiac problems, not being content to be examined, or inability to attend the educational sessions. Patients with the following conditions, which are known to interfere with or lead to the misinterpretation of HbA1c results, were excluded from participation: anemia, chronic renal disease, and/or hemoglobin variants [18].

Using the formula *n* = (*t*_*n*−1,*α*/2_ + *t*_*n*−1,*β*_/*d*)^2^*σ*^2^, power of 90%, error of 0.05, and study of Babazadeh et al., a sample of 24 people was calculated in each group, and 50 people were included in the study in order to increase the power of the study and compensate for possible dropout in each group [19].

**Figure 1:**
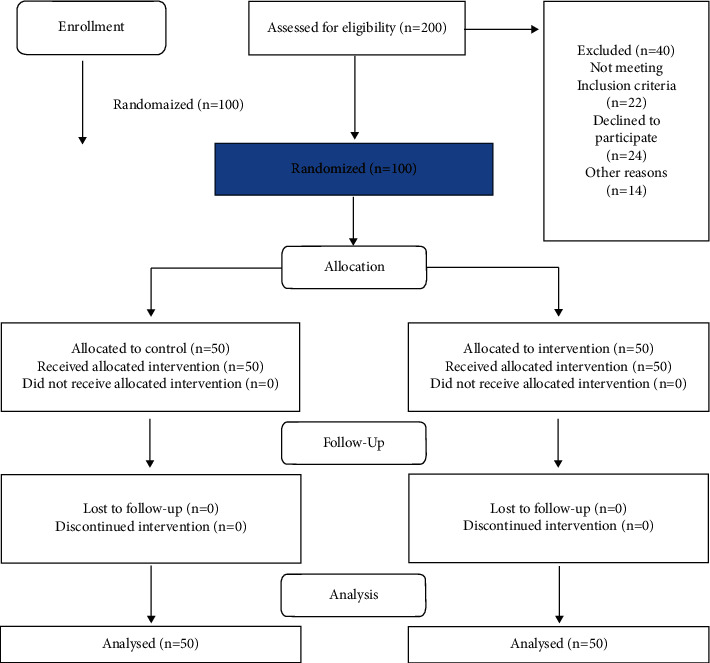
The study flow diagram.

At the end, from 150 remaining subjects, 100 patients have been chosen randomly. They were divided into two groups: experimental and control (50 patients for each group). The coin tossing method was used to allocate patients in the study groups. For the group that reached 50 people earlier, the sampling of that group ended, but the sampling continued for the other group. This way, the first person went to the experimental group, the second person went to the control group, and so on. In the current investigation, the used tool for gathering information is a questionnaire designed based on other similar studies [20–23] (Figure 1).

The first section includes questions about demographic information such as age, sex, education, job, duration of diabetes, and history of diabetes in family.

The second section includes questions related to the health belief model structures. There are 15 items for knowledge (the correct answer has a score of 1, and the incorrect answer has a zero score) (a sample question: “eye damage can be prevented by regular control of blood sugar”), 5 items about perceived susceptibility (such as “I may have optical problems, even though I regularly control my blood glucose level”), 5 items about the perceived severity (such as “if I get infected by optical problems caused by diabetes, I would become disabled and my life would have lots of problems”), 5 items about the perceived benefits (such as “if I refer to the optometrist regularly, I would not become blind”), 5 items about perceived barriers (such as the high costs of referring to the optometrist), 5 items about the self-efficacy (such as “I'm sure that I can have proper diet”), and 10 items for cues to action (such as “the optometrist advised me to take preventive measures against blindness caused by diabetes”). All questions are designed based on the standard five-point Likert scale, from “completely disagree” to “completely agree” (from 1 to 5 scores). About patients' performance in preventive behaviors against optical problems, 6 questions are asked (such as walking at least 3 times per week for 20 minutes, regular drug intake according to the instruction, referring to optometrists, having appropriate diet, glucose testing, and participating in educational sessions). All questions are designed based on the following: “yes” score = 1 and “no” score = 0. Before and three months after the educational intervention, the purpose of the study and how to complete the questionnaire were explained to the participants. Then, the questionnaires were distributed to the experimental and control groups and were completed by the subjects. The questionnaires were answered through self-reporting.

In order to determine the qualitative face validity of tools, a list of developed items was piloted to 30 type II diabetic patients with similar demographic, economic, and social characteristics. In this method, these people are interviewed face-to-face, and their opinion about the level of difficulty, appropriateness, and ambiguity is taken for each of the items. After modifying the items based on the opinions of these people, the next step was to determine the content validity of the questionnaire. In order to determine the content validity, the ideas of 12 specialists (out of research team), namely, 9 specialists in health education and promotion, 1 retina specialist, 1 endocrinologist, and 1 vital statistics specialist, have been employed. The necessary items have been selected and maintained based on Lawshe's table (based on the number of evaluators, i.e., 0.56 for 12 persons). In this study, Cronbach's alpha coefficient is used to determine the internal correlation in each of the subscales and the whole instrument. Cronbach's alpha represents the appropriateness of a group of items that measure a construct [24].

Cronbach's alpha coefficient was used to check the internal consistency, and values greater than 0.6 were considered acceptable. In this study, the calculated values for most of the items are higher than 0.70. The general consistency of research tools, by calculating Cronbach's *α*, is 0.88. The consistency of perceived susceptibility is 0.75, perceived severity is 0.84, perceived benefits is 0.78, perceived barriers is 0.81, self-efficacy is 0.78, and cues to action is 0.80. Because the calculated values of Cronbach's *α* for each of the studied structures are higher than 0.7, the consistency of tools is appropriately evaluated and confirmed. For ethical considerations, in addition to the approval of the ethical committee of Fasa University of Medical Sciences and diabetes center of Fasa city and subjects' consent, the aims, importance, and demands of performing this kind of study were revealed to the subjects, and they were ensured that their personal information would remain confidential.

Before educational intervention in both experimental and control groups and filling the mentioned questionnaire by subjects, the patients have been referred to a laboratory to have HbA1c test. HbA1c test is a diagnostic criterion for diabetes (DM) in the general population (the cutoff: HbA1c ≥ 48 mmol/mol (6.5%)) [25, 26].

After an overnight fast, blood samples were drawn to determine HbA1c levels [25].

Then, the educational intervention for the experimental group was performed in 8 educational sessions (in 50 to 55 minutes) by giving presentations, asking and answering questions, holding group discussions, and presenting educational films, images, and PowerPoint slides. In these sessions, subjects learned about diabetes and its effect on eye, the optical problems, the effect of having proper diet and regular medicine intake for preventing optical problems, the importance of participating in educational sessions, the importance of regular exercising in controlling blood glucose level, the importance of regular reference to optometrists, etc.

In one of the sessions, a 55-year-old man who has become blinded due to diabetes was invited to talk with subjects about his illness. Moreover, an optometrist was invited to examine the patients. At the end, educational booklets were given to the subjects of the experimental group, and a telegram group was provided for exchanging information. The experimental group received the training in eight sessions; each session lasted for 50 to 55 minutes, and two follow-up sessions were held one month and two months after the educational intervention. 3 months after the educational intervention, a questionnaire was filled out by the experimental and control groups, and the patients were referred to another HbA1c test. In this study, there were 2 observers, and the agreement index like kappa coefficient is equal to 0.76. In this study, the simple *P* value is used. In order to analyze the studied data, SPSS 22 software (SPSS Inc., IBM, Chicago, IL, USA), Chi-square, independent *t*-test, and paired *t*-test have been used. Data analysis was done by intention-to-treat method.

## 3. Results

In the current research, 100 diabetic patients attending the diabetes center of Fasa city are investigated. The average age of the experimental group is 45.28 ± 7.16, and that of the control group is 44.45 ± 7.24 years (*P*=0.125). The average duration of diabetes in the experimental group is 12.24 ± 3.18, and that of the control group is 12.90 ± 3.50 years (*P*=0.241). The independent *t*-test does not indicate any significant differences between two groups. Chi-square test shows that the experimental and control groups do not have any significant differences in some variables such as the educational level (*P*=0.155), job (*P*=0.155), family history of diabetes (*P*=0.364), and sex (*P*=0.216) (Table 1).

The obtained results revealed that, according to the independent *t*-test, before the educational intervention, there seemed to be no significant difference between the average scores of knowledge (*P*=0.08), perceived susceptibility (*P*=0.116), perceived severity (*P*=0.145), perceived benefits (*P*=0.107), perceived barriers (*P*=0.214), self-efficacy (*P*=0.212), cues to action (*P*=0.161), and eye care practice (*P*=0.170) between experimental and control groups. However, 3 months after the educational intervention, a significant difference has been observed (*P* < 0.05), and paired *t*-test showed that the average scores of knowledge, perceived susceptibility, perceived severity, perceived benefits, self-efficacy, cues to action, and eye care practice of experimental group enhanced and the average score of perceived barriers reduced (*P* < 0.05). Furthermore, in the control group, the average scores of knowledge, perceived susceptibility, perceived severity, perceived benefits, perceived barriers, self-efficacy, action guide, and eye care practice have no significant changes (*P* > 0.05) (Table 2).

The results of this study show that, according to the paired *t*-test, the average value of HbA1c in the experimental group reduced 3 months after the educational intervention (*P* < 0.001), while in the control group, there seemed to be no significant changes (*P*=0.134) (Table 3).

## 4. Discussion

Diabetic patients should be educated about preventive methods, treatment, and controlling their disease in order not to become infected by the side effects of diabetes. In order to prevent the optical problems of diabetes, diabetics must frequently refer to doctors; have proper diet, physical activities, and timely drug intake; and control their blood glucose level. Optical problems of diabetes are not specified for patients in early stages, and retinopathy observation is suggested for all diabetic patients [27].

Results indicate that the educational intervention causes the enhancement of studied patients' knowledge. This is consistent with the results of Dadkhah Tehrani et al. [28], Siminerio et al. [29], and Sadeghi et al. [30] studies. The reason of the increase of experimental group knowledge is the educational programs and booklets that subjects received about preventing blindness.

The results of the present research show that the values of perceived susceptibility and perceived severity (perceived threat) of experimental group have significant enhancement. This is consistent with the results of Alatawi et al. [31], Rees et al. [32], and Kouhpayeh et al. [33] studies. The educational intervention for the experimental group is performed in educational sessions by giving presentations, asking and answering questions, holding group discussions, and presenting educational films, images, and PowerPoint slides that increased the perceived sensitivity in people too. In one of the sessions, a 55-year-old man who has become blinded due to diabetes was invited to talk with subjects about his illness, which increased the perceived severity in people.

The results of this study show significant enhancement in the average score of perceived barriers in the experimental group. This is consistent with the results of Hartnett et al. [27], Sadeghi et al. [34], Kouhpayeh et al. [33], and Sheppler et al. [35]. It means that, after the educational intervention, the experimental group learned about the benefits of preventive behaviors against optical problems of diabetes and had fewer barriers against adopting these behaviors. In the current study, the presentation of education about diet and physical activities, the presence of an optometrist, the educational and motivational SMSs that patients received, and the educational booklets helped the increase ofperceived benefits and decrease perceived barriers dominating on barriers.

Providing educational booklets for diabetic patients, availability of an optometrist, follow-up contact after educational sessions, a telegram group for exchanging information, asking and answering questions, and presenting educational films and images caused the increase performance of eye care in the experimental group. The obtained results are in a good agreement with other studies [36–38].

The average score of cues to action after the educational intervention enhanced in the experimental group, indicating that the experimental group has proper internal and external motivations for adopting eye care behaviors. In the present research, the most important external cues to action of subjects are optometrists, diabetes center officials, and family members of participants. In the studies of Amini Moradi et al. [39], Malekmahmoodi et al. [40], and Sadeghi et al. [41], the educational intervention caused an increase of the average score of cues to action.

In the study of Van Eijk et al. [42], fear from blindness was one of the motivational factors in diabetic retinopathy observation, and the most important barrier was the lack of suggestions from healthcare officials. The results of Fortmann et al. [43] and Sheya et al. [44] indicated that diabetic patients with social support have better blood glucose control.

The average score of performance in the experimental group increased after the educational intervention. In this study, some behaviors such as walking and exercising, regular medicine intake, having proper diet, regular control of blood glucose level, and reference to optometrists are the eye care practices. According to the high scores of awareness, perceived susceptibility, severity, benefits, self-efficacy, and cues to action and the reduction of perceived barriers in the experimental group, the performance score has enhanced. The results of this research about performance are in a good agreement with the studies about self-care behaviors of type II diabetic patients [45], eye care of diabetic patients [46], dietary habits [47], walking of diabetic patients [22], medical regime of diabetic patients [48], eye care of diabetic patients [29], metabolic control [13], and controlling blood glucose level [49].

The results of the present research show that, after the educational intervention, the experimental group has significant reduction in HbA1c value. The results of this study agree with the results of Salinero-Fort et al. [50], Elabbassy et al. [51], and Shamsi et al. [14]. According to the high scores of awareness, perceived susceptibility, severity, benefits, self-efficacy, and cues to action and the reduction of perceived barriers in the experimental group, the performance score of HbA1c test has enhanced. One of the limitations of the present study is self-reporting of eye care practices such as medical intake and diet as it is not possible to observe the performance of subjects. In addition, the sample size in this study was very small, and the present results are related to participants who were referred to health centers of Fasa city, Iran; therefore, this study cannot be generalized to all diabetic patients. The investigation of HbA1c criterion for controlling blood glucose and the availability of an optometrist for diabetic patients are the positive points of this research.

## 5. Conclusions

The results of this study show that educating type II diabetic patients about eye care practice based on health belief model causes the enhancement of subjects' performance. Therefore, educational programs based on health education patterns and promotion for diabetic patients for preventing side effects caused by diabetes should be performed. All of diabetic patients should be referred to optometrists at least every year. Providing education through social media, especially television,about preventive measures against optical complications, such as having proper diet, regular medicine intake, and physical activities, is demanded.

## Figures and Tables

**Table 1:** Demographic information of studied subjects.

Variables		Experimental group		Control group		*P* value
		Number	Percentage	Number	Percentage	
Educational level	Illiterate	1	2	1	2	0.155
	Elementary	3	6	4	8	
	Guidance school	16	32	13	26	
	High school	18	36	21	42	
	University	12	24	11	22	
Sex	Female	28	56	30	60	0.216
	Male	22	44	20	40	
Job	Employee	8	16	10	20	0.105
	Self-employed	4	8	6	12	
	Farmer	1	2	1	2	
	Housewife	19	38	18	36	
	Others	18	36	15	30	
Family history of diabetes	Yes	12	24	10	20	0.364
	No	38	76	40	80	

**Table 2:** Comparison of average scores of HBM components of studied subjects before and 3 months after the educational intervention in experimental and control groups.

Variables	Groups	Before educational intervention	3 months after educational intervention	Paired *t*-test
Awareness (0–15)	Experimental	6.22 ± 2.84	11.31 ± 2.45	0.001
	Control	7.09 ± 2.50	7.28 ± 2.47	0.128
	Independent *t*-test	0.080	0.001	
Perceived susceptibility (5–25)	Experimental	8.24 ± 3.25	19.24 ± 2.81	0.001
	Control	8.42 ± 3.28	9.06 ± 3.18	0.126
	Independent *t*-test	0.116	0.001	
Perceived severity (5–25)	Experimental	8.42 ± 2.18	18.26 ± 2.28	0.001
	Control	8.17 ± 2.24	8.48 ± 2.84	0.0131
	Independent *t*-test	0.145	0.001	
Perceived benefits (5–25)	Experimental	8.71 ± 2.13	18.36 ± 2.71	0.001
	Control	9.04 ± 2.16	10.01 ± 2.13	0.184
	Independent *t*-test	0.107	0.001	
Perceived barriers (5–25)	Experimental	19.36 ± 2.53	8.25 ± 2.50	0.001
	Control	18.87 ± 2.45	17.40 ± 2.44	0.155
	Independent *t*-test	0.214	0.001	
Self-efficacy (5–25)	Experimental	8.23 ± 2.18	18.44 ± 2.18	0.001
	Control	8.45 ± 2.10	9.12 ± 2.02	0.224
	Independent *t*-test	0.212	0.001	
Cues to action (10–50)	Experimental	18.22 ± 4.14	36.87 ± 4.45	0.001
	Control	17.91 ± 3.85	18.34 ± 3.70	0.241
	Independent *t*-test	0.161	0.001	
Eye care practice	Experimental	2.16 ± 0.79	4.24 ± 1.112	0.001
	Control	2.24 ± 0.48	2.27 ± 0.57	0.148
	Independent *t*-test	0.0170	0.001	

**Table 3:** Comparison of average values of HbA1c in experimental and control groups before and 3 months after educational intervention.

Variables	Groups	Before educational intervention	3 months after intervention	Paired *t*-test
HbA1C	Experimental	8.75 ± 1.82	7.25 ± 1.60	*P* < 0.001
	Control	8.79 ± 1.78	8.70 ± 1.81	*P*=0.134
	Independent *t*-test	*P*=0.105	*P* < 0.001	

## Data Availability

The datasets used and/or analyzed during the current study are available from the corresponding author upon reasonable request.

## Abbreviations

HBM: Health belief model.
